# Supramolecular Nanostructures for Vaccines

**DOI:** 10.3390/biomimetics7010006

**Published:** 2021-12-29

**Authors:** Ana Maria Carmona-Ribeiro

**Affiliations:** Biocolloids Laboratory, Departamento de Bioquímica, Instituto de Química, Universidade de São Paulo, Avenida Professor Lineu Prestes, 748, Butantan, São Paulo CEP 05508-000, SP, Brazil; amcr@usp.br

**Keywords:** vesicles, liposomes, bilayer disks and fragments, cationic lipids and polymers, biocompatible polymers, niosomes, nanoparticles, nanogels, hydrogels, adjuvants for humoral and cellular immune responses

## Abstract

Although this is an era of pandemics and many devastating diseases, this is also a time when bionanotechnology flourishes, illuminating a multidisciplinary field where vaccines are quickly becoming a balsam and a prevention against insidious plagues. In this work, we tried to gain and also give a deeper understanding on nanovaccines and their way of acting to prevent or cure cancer, infectious diseases, and diseases caused by parasites. Major nanoadjuvants and nanovaccines are temptatively exemplified trying to contextualize our own work and its relative importance to the field. The main properties for novel adjuvants seem to be the nanosize, the cationic character, and the biocompatibility, even if it is achieved in a low dose-dependent manner.

## 1. The Importance of Nanoadjuvants for Vaccines

In this pandemics era, the exponential growth of nanobiotechnology certainly benefits the development of nanovaccines, displaying resourceful alternatives for controlling and hopefully eradicating infectious diseases [1,2,3,4,5,6]. The early models for vaccination using attenuated pathogens offered the antigens and the pathogen molecular patterns (PMPs) able to properly activate the antigen presenting cells (APCs), however, many modern vaccines carry purified antigens so that adjuvants and stimulators become essential to trigger activation of APCs. Against many diseases such as cancer, tuberculosis, mycosis, malaria, and parasites infestation, vaccines have to enhance both antibody production and cellular immunity [7].

Most commercially available vaccines promote antibody and Th2 response and are able to fight invading microorganisms that do not enter the host cells. However, against intracellular infections and cancers, both cellular and humoral immune responses are essential [8]. The CD8+ T-cells respond to infection or transformation of nucleated cells expressing major histocompatibility complex (MHC) class I and, upon differentiation into cytotoxic T-lymphocytes (CTLs), kill target cells directly; in addition, CD8+ T-cells release chemokines and cytokines that call more effector cells, such as the neutrophils and the macrophages [8].

Because most antigens are negatively charged, electrostatics guarantees that cationic nanoadjuvants properly adsorb antigens, yielding nanometric sizes for the adjuvant/antigen assemblies that are not retained at the site of injection, avoid physiological barriers, and are rapidly drained to the lymph nodes rich in dendritic cells [3,9,10,11,12,13]. Nanoadjuvants can be obtained from a variety of lipids, polymers, or lipids, and polymers mostly as bilayer vesicles [14,15], open bilayer disks [16,17,18,19,20], lipid nanoparticles [21], or hybrid nanoparticles [4,5,22,23,24,25,26]. For example, the molecular geometry of dioctadecyldimethylammonium bromide (DODAB), a cationic and synthetic double-chained amphiphile, favors its self-assembly in water solution as bilayers either closed (large vesicles) or open (bilayer fragments or disks) [15,27]. The latter, from now on called DODAB BF, displays nanometric sizes (<100 nm), have hydrophobic defects and fuse upon salt-induced screening of their surface potential [28]. DODAB bilayers are very versatile, adsorbing several biomolecules: proteins [14,17,29,30], peptides [25,31,32], oligonucleotides [18,33], mononucleotides [34,35], and nucleic acids [36,37], and also enhancing cellular immune responses to antigens, though yielding poor humoral responses [14,17,18,22]. DODAB assemblies also interact with negatively charged particles such as polystyrene sulfate latexes [22,25,38,39,40] or silica [41,42,43,44]. Optimal bilayer deposition led to the description of supported DODAB bilayers on polystyrene sulfate (PSS) nanoparticles [22,25,40] or silica for presentation of adsorbed antigens [43,44]. The good miscibility of DODAB with the biocompatible polymer poly (methylmethacrylate) (PMMA) [23,45] led to the synthesis of polymeric PMMA nanoparticles by emulsion polymerization of methylmethacrylate (MMA) in the presence of DODAB BF, which produced the hybrid, cationic, nanometric and biocompatible PMMA/DODAB NPs [26]. These NPs combined well with oppositely charged ovalbumin (OVA) and induced a mixed Th1-Th2 immune response [46].

For use in humans, there are few adjuvants [47]. Commercially available vaccines largely promote antibody and Th2 responses [48]. In the US, there are aluminum salts (alum) added of monophosphoryl lipid A, a toll-like receptor 4 agonist [48], whereas, in Europe, there are the virosomes, the virus-like particles (VLPs), and the oil-in-water emulsions [49]. A good example of vaccine with VLPs as adjuvants is a prophylactic vaccine against infections by the human papilloma virus (HPV) that is able to prevent cervical cancer [50].

Strong Th1 response in animals has been provided by viral constructs [51,52], DNA- [53,54,55], and mRNA-based vaccines [54,56,57]. These vectors promote the process of cross-presentation in APCs, meaning that vaccine-encoded peptide fragments are released in the cytosol and presented by MHC-I in APCs [6,58,59]. DNA- and mRNA-based vaccines have success for veterinary use [60]. However, efficacy, safety, and tolerability remains to be established for humans, despite of the use of these technologies in coronavirus vaccination [61,62].

Bacterial toxins, particulates, plant derivatives, and pathogen-associated molecular patterns (PMPs) are some examples of adjuvants [63,64]. Adjuvants belong to two groups: they are immunopotentiators or they are delivery systems according to their mechanism of action [48,65]. Delivery systems prevent antigen degradation and promote uptake of antigens by APCs [65], whereas immunopotentiators act as agonists of pathogen recognition receptors (PRRs) [48]. Efficient adjuvants may result from combinations of delivery systems and immunopotentiators [66,67].

In this review, supramolecular assemblies yielding nanostructured materials able to combine well with antigens to potentiate the immune response are overviewed. Nanoparticles, vesicles, liposomes, bilayer disks, niosomes, and hydrogels are evaluated from the perspective of efficient antigen presentation in novel nanovaccines.

## 2. Molecular and Supramolecular Assemblies for Vaccines and Beyond

**Figure 1:**
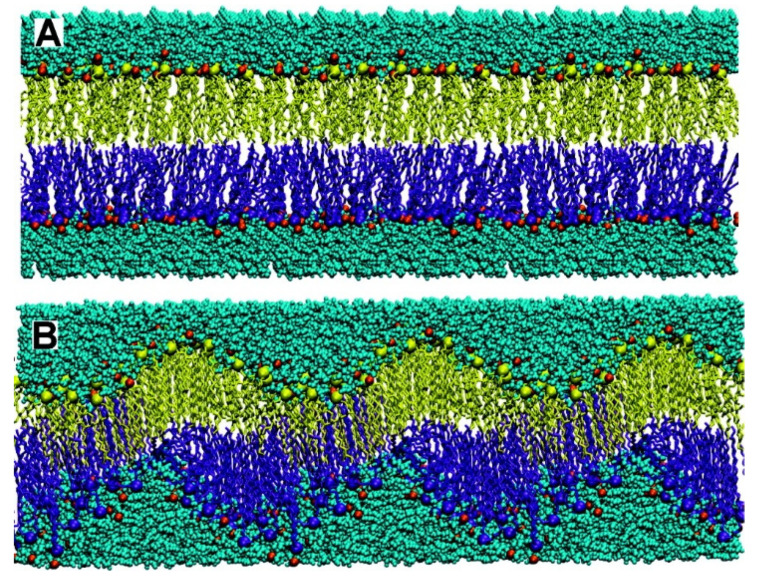
The dynamics of the self-assembly process simulated in the computer for dioctadecyldimethylammonium bromide at 25 °C at times zero (**A**) and 90 ns (**B**). Reprinted with permission from [80]. Copyright 2010 American Chemical Society.

Molecular assemblies based on non-covalent, physical intermolecular interactions have been extensively explored in drug and vaccine delivery [16,67,68,69,70,71]. For example, amphiphiles such as lipids and surfactants self-assemble in water solution to yield a variety of useful nano- or micro-structures such as micelles, bilayer membranes, inverted phases, vesicles, nanodisks, etc.; type of assembly can be predicted from theory and molecular geometry [72,73]. Intermolecular interactions can be attractive and/or repulsive depending not only on the chemical structure of molecules involved but also on composition of the intervening medium [45,74,75]. Adsorption of counter-ions onto a cationic bilayer can modify the type of bilayer assembly. NaCl added to DODAB BF promoted fusion and appearance of large and closed bilayer vesicles in the dispersion and appearance of hydrophobic defects in the bilayer. Monovalent salt at a moderate concentration was reported to fuse cationic bilayer fragments [15,28,76,77], with induction of hydrophobic defects in the bilayer [78]. When the electrostatic repulsion is high, as in pure water or at low salt concentrations, interdigitation can take place, allowing some relaxation of the electrostatic repulsion between adjacent DODAB molecules in the bilayer. Adhesion between the cationic bilayers may occur due to interdigitation [79]. Computer simulations along time for the DODAB self-assembly in water dispersions [80], differential scanning calorimetry and scattering of X-rays in the subgel phase [81] evidenced consistently the intra-bilayer tilting and the occurrence of hydrophobic defects. Figure 1 illustrates molecular dynamics simulations for the self-assembly of the cationic lipid DODAB.

The importance of intermolecular interactions in determining structure and function of supramolecular assemblies made by men or found in nature is paramount. For example, biological supramolecular assemblies such as complexes of nucleic acids and histones occur in the chromatin of eukaryotic cells [82,83]. Surface electrostatic potential and less predictable hydrophobic contacts drive the DNA–protein interactions. The recognition of specific DNA sequences by proteins depends on the formation of hydrogen bonds with specific bases, primarily in the major groove, and on sequence-dependent deformations of the DNA helix. From analysis of the three-dimensional structures of protein–DNA assemblies, arginine residues attached to narrow minor grooves was often the mode for protein–DNA recognition; narrow minor grooves increase locally the DNA negative electrostatic potential thereby attracting positively charged regions on proteins [82].

**Figure 2:**
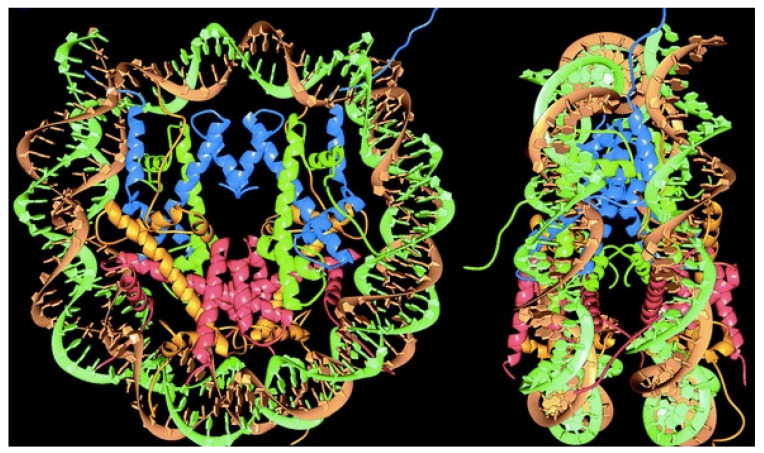
Nucleosome particle showing the histones core surrounded by DNA. Reproduced with permission from reference [84].

After crystallites of nucleosomes, the core particles of chromatin, were obtained, X-ray diffraction revealed their crystal structure at 2.8 angstroms resolution, a major achievement in 1997 [84]. Figure 2 shows the organization in the nucleosome particle; 146 base pairs of DNA helix surround the histones, representing a way of compacting long DNA chains of the chromatin in eukaryotes; linear DNA compaction thereby occurs by a factor of 30–40 [84]. The nucleosomes further associate into higher-order assemblies linked by the linker histone H1 so that DNA bends and forms high ordered helixes shaped by the histones [85]. The nucleosome is responsible for packaging DNA within the nucleus, largely determining DNA accessibility.

**Figure 3:**
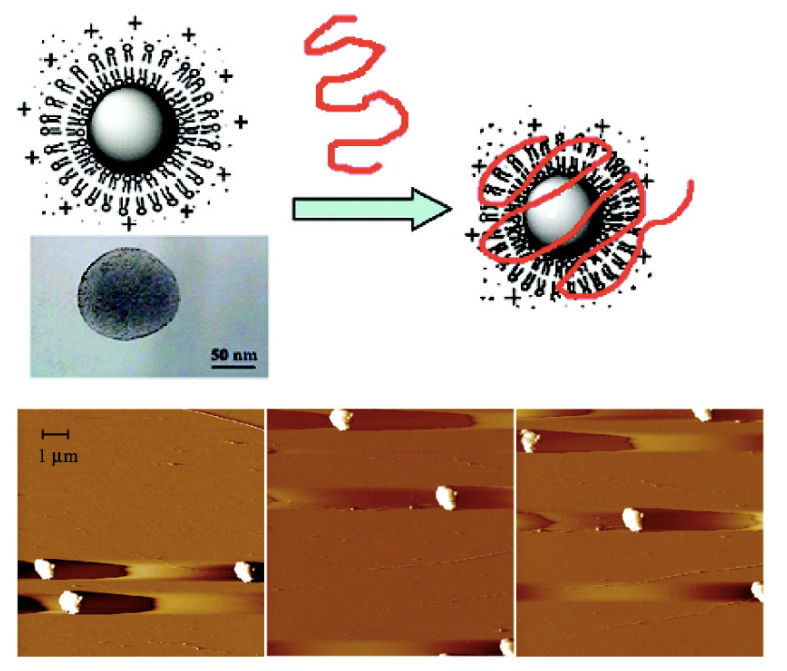
Scheme and AFM micrographs for PSS/DODAB/T2-DNA dispersions at charge neutralization. They display nucleosome-mimetic micrometric assemblies. One should notice some isolated PSS/DODAB nanoparticles and the long T2 DNA chain on the top right corner of the AFM micrograph on the left. Reproduced with permission from reference [36]. Copyright 2008. American Chemical Society.

Similarly to DODAB supramolecular assemblies, characteristics of nucleosomes also depend on physico-chemical properties of their environment such as ionic strength and divalent-ion concentration, and on histone or DNA-modification and/or DNA state [86]. For assemblies in vitro, variable nucleosome inter-spacing was related to variable electrostatic attraction between DNA and histones; if the salt concentration during the assembly process increased, the repeat distance between nucleosomes also increased [87,88]. The compacting effect of cationic NPs on long-chained bacteriophage DNA was reconstituted using supported cationic bilayers on polymeric nanoparticles; these nucleosome mimetic systems were available over a range of nanosizes for the primary polymeric particles surrounded by the supported cationic bilayer; this opened new possibilities for a variety of DNA- or mRNA-based nanovaccines [36]. Figure 3 shows images of supramolecular assemblies mimicking nucleosomes; they assembled from cationic nanoparticles and giant bacteriophages DNA under conditions of charge neutralization; in the same micrograph, some single nanoparticles and isolated DNA molecules could also be seen, although the majority of supramolecular assemblies corresponded to electrostatically driven associations between anionic DNA molecules and cationic PSS/DODAB NPs [36]. Another important feature was the compaction of the long DNA chains by the cationic NPs, imparting a truly nucleosome mimetic character for the assemblies.

**Figure 4:**
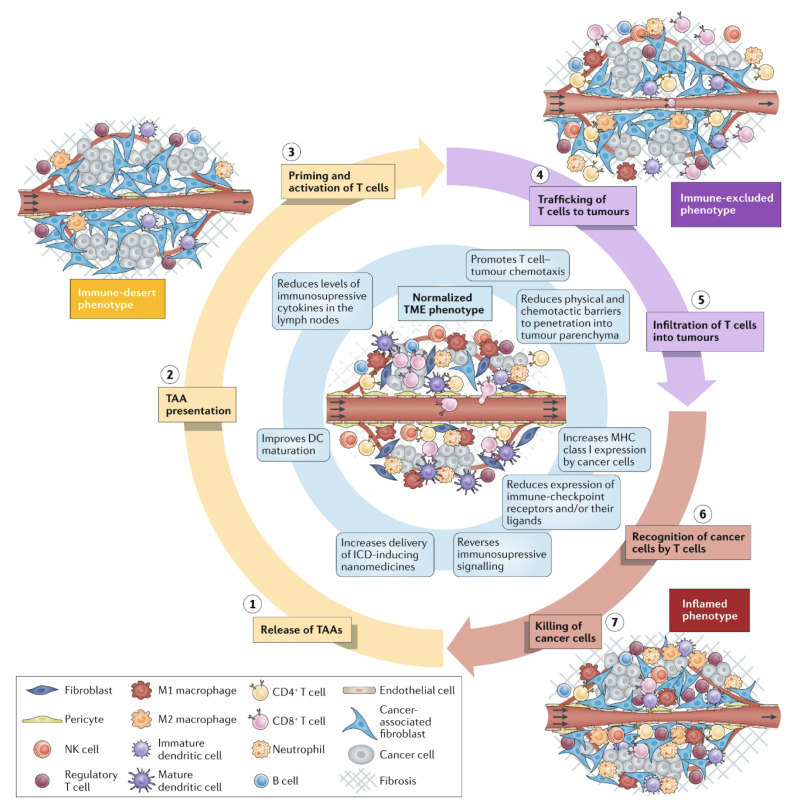
Possible tumor microenvironment phenotypes (TME) in cancer as compared to the normalized one. Combined medicines in nanoformulations, such as normalizers of TME and anti-cancer drugs, can advantageously penetrate the tumor mass as compared to microformulations. Abbreviations are DC for dendritic cell; ICD for immunogenic cell death; NK for natural killer; TAA for tumor-associated antigen. Reprinted with permission from [94].

Cancer immunity evolves in two steps: the preparatory and the effector ones [89]. The preparatory step takes place in the LN with release and presentation of cancer antigens for priming and activating T cells. In the next step, T cells reach the tumor mass, trying to recognize and kill the cancer cells in the tumor. However, the tumor tissue and microenvironment can be immunosuppressive due to low immunogenicity of tumor cells, presence of immunosuppressive cytokines, and biological barriers hampering chemotherapeutic agents and immunotherapies to reach the tumor cells [90,91]. Therefore, nanotechnology is needed for surmounting these key biological barriers and effectively deliver chemo- and immune-therapeutic agents and vaccines to their sites of action; sensitive antigens easily degradable in the physiological medium can be protected using nanotechnologies able to increase their half-lives, minimize systemic toxicity, and promote their delivery to APCs in the LN [91]. Nanostructures such as NPs [5,17,43,92], bilayer fragments (BF) [4,12], or peptide supramolecular assemblies [93] become drained directly to the LNs, performing well in the preparatory step [89]. In the effector step, activated T cells should infiltrate the tumor, and stable, long-circulating, and targeted nanostructures could help T cells infiltration improving the outcomes of immunotherapies, which may give a low response rate due to the T-cell poor infiltration in the tumor tissue. Despite the approval of nanomedicines for cancer treatment, the low observed survival was possibly due to abnormal phenotypes of the tumor microenvironment (TME); nanostructures should incorporate anticancer drugs, drugs for improving tumor perfusion, and others to return TME to normality [94]. The extracellular matrix (ECM) produced by cancer associated fibroblasts (CAF) supports cancer cells expressing α-smooth muscle actin and fibroblast activation protein (FAP), both upregulating the expression of other functional cell surface proteins like platelet-derived growth factor receptor β and the insulin-like growth factor receptor II; activated fibroblasts could benefit from delivery of nanoparticles carrying drugs, especially in liver cancer to downregulate growth factors [95]. Figure 4 reproduced from reference [94] illustrates the tasks ahead for nanomedicines: they should also enable normalization of abnormal TME phenotypes, such as the one restricting penetration of cells for immunotherapy (in purple) and the one with inflammatory character (in red); the normal TME appears centralized in blue. The defective vasculature in cancer tissue can not only reduce oxygen supply, but also hampers the penetration of anti-cancer cellular immunity; drugs and normalizers of the vasculature and drugs to prevent the production of dense extracellular matrixes by cancer associated fibroblasts would possibly normalize the TME by improving perfusion, oxygen supply and drug delivery of cytotoxic and anti-inflammatory chemotherapeutics. Importantly, responses to immunotherapy increased when TME normalization approaches were applied [94].

Having recognized the importance of formulating drugs and vaccines in nanostructures, here we present several instances of nano-vaccines delivery based on surfactants, lipids, polymers, biopolymers, proteins, etc. Self-assembled vehicles discussed are vesicles, liposomes, niosomes, bilayer fragments or disks, nanoparticles, and hydrogels.

## 3. Vesicles, Liposomes, Lipid Nanoparticles, Disks, and Niosomes

In this section, meaningful examples of vaccine delivery using self-assembled nanostructures are discussed and major research areas in need of vaccines are pointed out.

**Figure 5:**
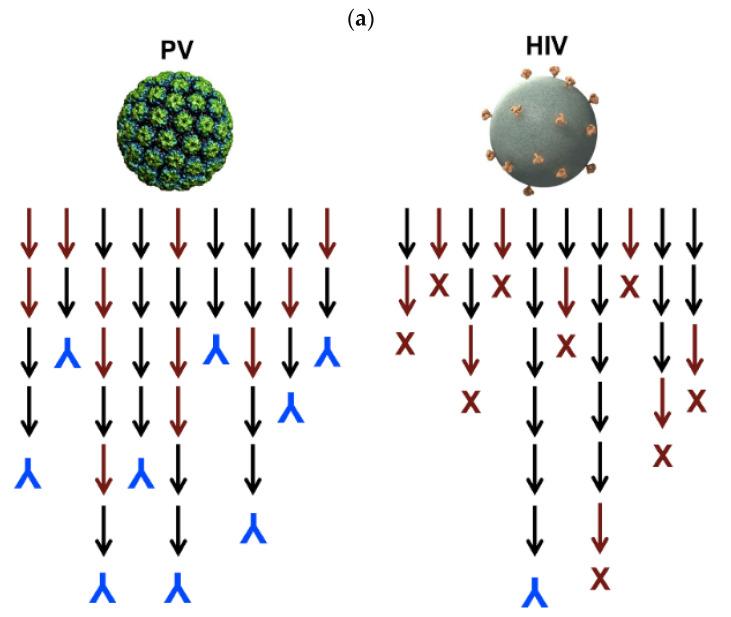

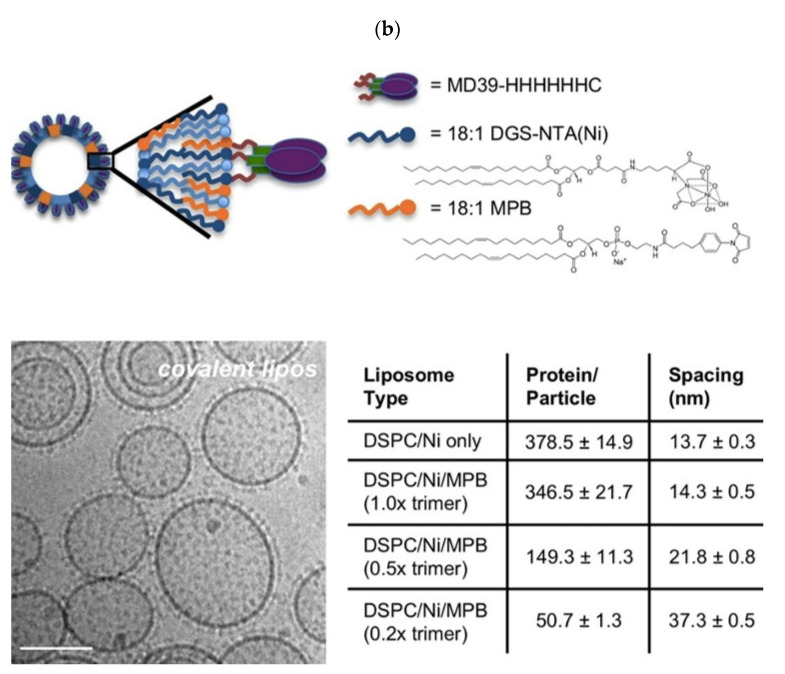
(**a**) Compared densities of spike proteins in papilloma virus (PV) and HIV able to elicit neutralizing antibodies (in blue). Reproduced from [96]. (**b**) Increasing the density of HIV antigens on liposomes. The HIV antigens were the MD39 trimmer covalently linked to the liposomes. On the cryo-electron microscopy micrograph, liposomes display the trimmers in a regular manner (bar  corresponds to 100 nm); on the table, trimmer densities and spacing in between them depends on coupling strategy and trimmer concentration used for coupling [97].

A HIV vaccine yielding long-lasting anti-HIV antibodies is not yet available. The immunodeficiency typical of HIV has its basis on the scarce numbers of trimmer spike proteins on the virus; this efficiently prevents IgG bivalent binding, meaning low elicitation of neutralizing antibodies; there are only 14 HIV spikes per virus [96]. Interesting combinations between antigen NPs and liposomes enhanced specific humoral responses against HIV. In a possible vaccine strategy, liposomes were employed to increase the density of exposed BG505 MD39, which is a gp140 envelope trimmer; covalently linked trimmers with good orientation were exposed by the liposomes at high densities; in this case, the nanoparticles attached to the liposomes were the trimmers; immunization with avid MD39-specific IgG antibodies in serum was thereby achieved [97]. Figure 5a illustrates the low density of trimmeric protein spikes on HIV and their implications in vaccine formulation, whereas Figure 5b shows the design of the liposomal formulation proposed to improve HIV antigen presentation at high densities [96,97]. Figure 5a evidenced the contrast between high-density and low density epitope display for papilloma virus (PV) and HIV, respectively, as reproduced from [96], whereas Figure 5b shows a liposomal formulation for displaying the gp140 trimer MD39; one should notice that the bar on the micrograph corresponds to 100 nm [97]. Liposomes in the electron microscopy micrograph eventually occur as concentric and closed multi-bilayers with a large variability in size, however there was a very regular distribution of the trimeric lipoprotein on the bilayers measured as the spacing between trimers on the bottom right portion of Figure 5b.

The spike protein of SARS-CoV-2 is the first point of virus contact with the cell to be invaded; for invasion, this viral protein binds to a host receptor. In order to prevent virus entrance in the host cell, it is possible to raise neutralizing antibodies able to attach to the receptor-binding domain of the viral spike protein; it is even possible to make a selection of the best antibodies, such as those recently found and named S2H97 and S2E12 [98]. The S2H97 antibody was able to neutralize several viruses belonging to the coronavirus family as well as coronavirus mutants; in vivo, hamsters treated with this antibody, and then infected with the virus two days after treatment, showed a serum decrease in viral RNA concentrations of more than ten thousand times as compared to the untreated hamsters [98]. S2H97 displayed a large breadth and resistance to escape, possibly encompassing neutralization of future mutants due to its broad capability to treat infections by several viruses belonging to the coronavirus family; patients recovered from coronavirus infection did not have antibodies able to compete with S2H97 for neutralizing the virus, meaning low probability of occurring mutants with changes in the epitope where S2H97 binds [98,99]. The S2E12 neutralizing ability was not as broad as the one of the S2H97, but its potency was also considerable; virus mutants escaping neutralization by S2E12 were not able to bind to the receptor of the host cell and therefore could not replicate, making their outbreaks unlikely [98]. S2E12 displayed a modest breadth against the virus of the coronavirus family and could not be ruled out, eventually becoming applicable to neutralize infections by coronavirus mutants or other members of the coronavirus family [98]. Importantly, in sub-Saharan Africa, the cross-reactivity in serum against the coronavirus was recently determined and associated to reduced figures of infections and deaths [100].

Despite the challenging requirement of messenger–RNA protection in vivo due to possible degradation by lytic enzymes, mRNA vaccines have been successfully formulated with nanoparticles made of lipids (LNPs); using several administration routes, these vaccines yielded high antigen production from images in vivo [101]. For example, LNPs safely and efficiently carried BNT162b2 mRNA Covid-19 vaccine developed and funded by Pfizer [62]. Although several formulations are available, these LNPs made of an amino lipid, phospholipid, cholesterol, and a poly (ethylene glycol)-lipid conjugate have been considered the most advanced ones [102]. LNPs were nanostructures with 70 to 100 nm in diameter and similar to those used for formulating small interference RNA [103]. The principles for designing an optimal mRNA lipid nanoparticle vaccine and major types of LNPs were recently reviewed [104,105]. Recently, the need for an additional improvement in m-RNA vaccines was pointed out: the optimization of the vaccines’ stability at low temperature [106].

**Figure 6:**
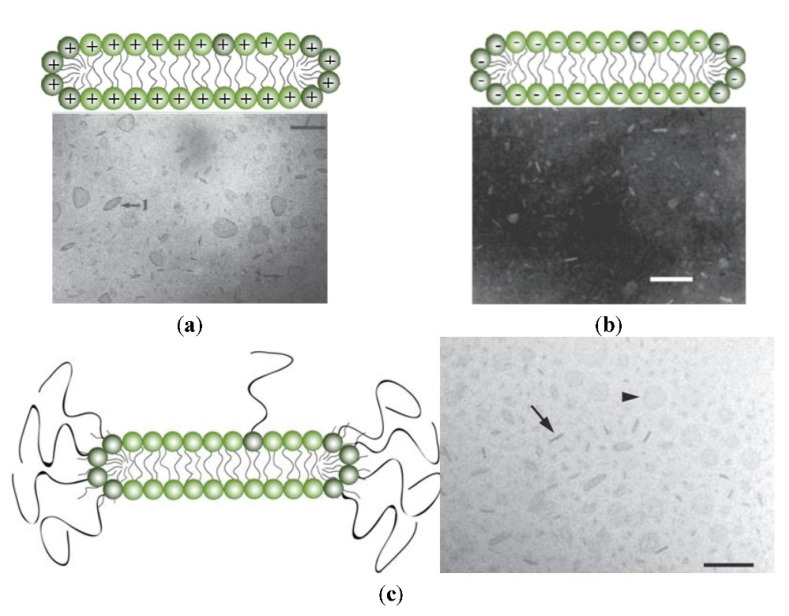
Schemes for cross sections of lipid bilayer fragments or disks and their respective micrographs. On (**a**), dioctadecyldimethylammonium bromide bilayer fragments or disks seen edge on **1** or face on **2** from cryo-transmission electron microscopy; bar is 100 nm. Adapted with permission from [20]. Copyright 1995 American Chemical Society. On (**b**), sodium dihexadecylphosphate bilayer fragments or disks seen after negative staining by transmission electron microscopy: scale bar is 100 nm. Adapted with permission from [109]. Copyright 1991 American Chemical Society. On (**c**), composition of phospholipid, cholesterol, and ceramide conjugated to poly (ethylene glycol) (35:40:25 mol%) seen edge on (arrow) or face on (arrow head) by cryo-TEM; scale bar is 100 nm. Reprinted from [110] with permission from Elsevier, Copyright 2011.

Instead of using complicated compositions and expensive lipids, an unexplored though promising strategy is formulating the antigen with bilayer disks made of DODAB; they are positively charged, chemically stable, display colloidal stability in water due to the electrostatic repulsion, and readily adsorb oppositely charged nucleic acids or protein antigens [16,17,19,79,107,108]. Furthermore, DODAB as immunoadjuvant elicits a potent cellular immune response so often required against several pathogens and cancer, despite DODAB dose-dependent toxicity [3,4,12,46]. Figure 6 shows some LNP disks made from cationic lipid DODAB [20] (Figure 6a), anionic lipid dihexadecylphosphate DHP [109] (Figure 6b), or from a neutral composition of several lipids including PEGylated-lipids [110] (Figure 6c).

Niosomes are non-ionic surfactant vesicles consisting of one or more than one closed bilayer delimiting inner water compartment(s) [111,112]. First described by Uchegbu and Florence as similar to liposomes, they could be obtained when surfactants were added to water [113] and their utility for antigen presentation and vaccines delivered by the oral route revealed their potential as mucosal adjuvants able to carry not only protein antigens, but also genetic material [114,115]. Compared to phospholipids/cholesterol liposomes, niosomes were chemically more stable and only slightly more leaky than the liposomes tested for determining permeability of the fluorescent compound calcein; permeability of niosomes towards KCl was also higher than the one displayed by the liposomes, thus niosomes were more leaky self-assembled bilayers [111].

Aiming at an oral vaccination against tetanus, the stability of niosomes carrying the tetanus toxoid was improved by adding O-palmitoyl pullulan to the composition; the polysaccharide moiety imparted stability to the formulation in a liquid that had a pH of about 1.5 and was similar to the gastric juice. These loaded niosomes, delivered orally, yielded a higher humoral response than the controls and high mucosal IgA titers, showing their suitability for oral vaccine delivery [112].

## 4. Nanoparticles

In a major, highly recommended review article, Irvine and coworkers considered that biotechnology combined with the nanomaterials science could bring about safer and more efficient vaccine formulations with major roles for the nanoparticles (NPs) [116]. In a fast moving field encompassing cancer nanotechnology [50,89,90,95,117], vaccinology against pathogenic viruses [61,62,96,97,98,99,100,106,118], and microbes such as bacteria [19,108,112,119,120], fungi [119], and parasites [22,120,121,122,123,124], elegant nanomaterials that are difficult to scale up for commercialization cannot become useful in clinics [116,125].

**Figure 7:**
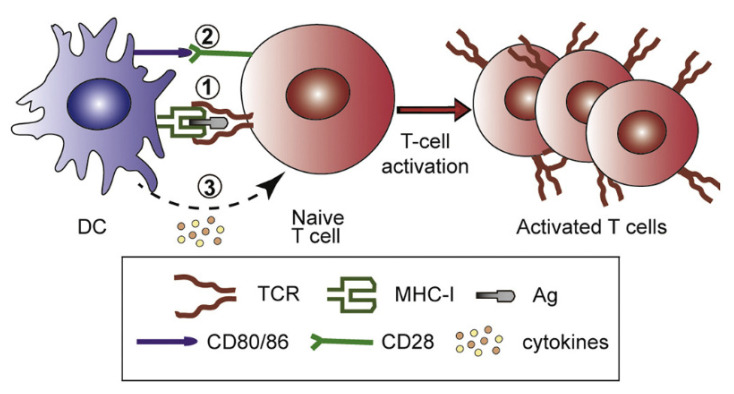
Events for T-cell activation. Reprinted from [8,126]. Reprinted from [126] with permission from Elsevier, Copyright 2017.

Synthetic nanoparticles for cancer vaccines have to fulfill several tasks: tumor antigens and stimulators have to be delivered to APCs in LN so that inside the APC, antigen escape from the lysosomes to the cytosol defines antigen cross-presentation via major histocompatibility complex I (MHC-I), promoting the cellular immunity represented by the cytotoxic T lymphocytes (CTL) [117]. Figure 7, taken from references [8,126], illustrates the sequence of events for T-cell activation, namely, Ag delivery to DC in the LN, Ag intracellular traffic in DCs to their cytosol, and cross-presentation by MHC-I; secretion of stimulatory molecules and cytokines; type-I interferons stimulate the differentiation of naïve CD4 + T cells into Th1 subtype, whereas IL-4 leads to Th2 subtype. Against cancer and intracellular infections such as in malaria [121] and tuberculosis [126,127,128,129], cellular immune responses are essential.

The problem of delivering Ag to DCs involves its transport to DC-rich areas such as the LN, its binding to DCs and its internalization by DCs for Ag processing and presentation [130]. Clearance of NPs smaller than 5 nm from the blood is very fast so that they bypass the LN. The effect of particle size on the delivery route for NPs to LN was described: NPs larger than 200 nm mean diameter largely depended on dendritic cells transportation and entered the LNs after 18 h; NPs with diameters smaller than 200 nm entered LN within 2–3 h and were drained by the lymphatic vessels directly to the LN, independently of DC-transportation [9]. As the second route is much faster, nanovaccines have been developed using NPs with diameters smaller than 200 nm and larger than 5 nm.

Synthetic nanoparticles that mimic viruses are not only safe and efficient, but also advantageous from the point of view of strengthening the immune response [131]; virus-mimetics can benefit vaccine design and was recently reviewed [132]. Viruses are regular biological nanoparticles, all of the same size, often displaying several copies of antigenic spike proteins in a regular manner surrounding its nanoparticle structure, these properties common to several viruses should be copied in virus-mimetic vaccinology.

DNA synthesis for encoding several antigens of bacteria allowed the expression of all the antigens for attachment to a lipid core peptide adjuvant and self-assembly based on the lipid core; 40 nm diameter NPs spontaneously formed upon addition of the lipoproteins to the buffer, thereby antibodies against all antigens could be produced in mice without any further need of adjuvants [133].

The antimicrobial peptides that occur in nature [134] motivated a whole new area of peptidomimetics that is heavily based on chemical synthesis and library screening for selecting optimal antimicrobial activity [135]; for example, the LptD is a β-barrel protein in bacteria essential for bacteria outer membrane synthesis; the peptidomimetic assemblies mimicking LptD at nanomolar concentrations blocked outer membrane synthesis of *P. aeruginosa* [136].

**Figure 8:**
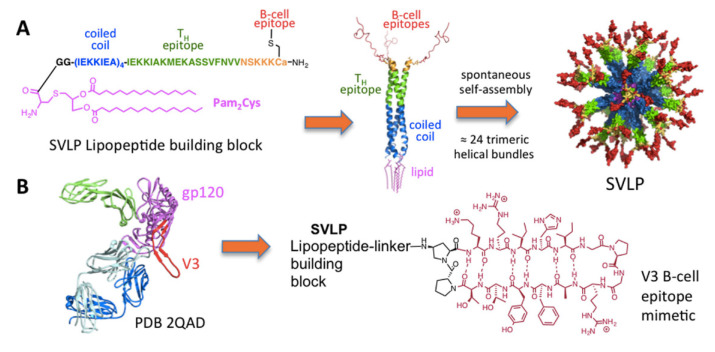
(**A**) Chemical structure and self-assembly of lipopeptides with epitopes to stimulate B cells (orange) and T cells (green) yielding synthetic virus like particles (SVLP). (**B**) SVLP aiming at eliciting antibodies against HIV. Reproduced from reference [137] with permission from American Chemical Society Copyright 2017.

The design of supramolecular synthetic vaccines has also been using robust chemical synthesis to mimic antigen epitopes [137]. Synthetic nanoparticles similar to viruses or synthetic virus-like particles (SVLPs) prepared using supramolecular chemistry can display B- and T-cell epitopes and ligands for pattern recognition receptors; this would achieve the optimal surface properties for efficient dendritic cell-mediated delivery of B-cell and T-cell epitopes, and also agonists for pattern recognition receptors, into lymph nodes. In addition, the multiple presentation of the epitope mimetics on the surface of the nanoparticle was highly immunogenic, triggering strong epitope-specific humoral immune responses that target the pathogen causing the infection. Figure 8 shows some hybrid lipid–peptide covalent constructions achieved by chemical conjugation with applications against pathogenic bacteria or in vaccine design. Chemical conjugation strategies for the development of protein-based nanovaccines have been reviewed [126].

Nanotechnologies impact the vaccine field. Through decoration of immunogens as multiple copies on nanoparticles, improved humoral immunity can be achieved, however scaling-up production of these nanovaccines is still a major draw-back for their use in clinics. Trying to circumvent this issue, an interesting approach was the delivery of synthetic DNA by electroporation to obtain in vivo the synthesis of the multivalent nanoparticles; the self-assembly of multiple HIV antigens to yield a nanoparticle took place in vivo, inducing higher antibody titers than the monomers and also eliciting cellular immunity in contrast to the recombinant protein nanovaccine; similar results were obtained using hemagglutinin DNA nanovaccine, where the nanovaccine with multiple copies of the epitopes of interest was produced in vivo yielding protection against influenza in mice [138]. Recently, a similar approach using DNA-launched vaccines was applied for suppression of melanoma tumors: multiple copies of Gp100 and Trp2 epitopes in nanoparticles assembled in vivo induced stronger immune response and CD8+ T cells immunity than the corresponding DNA monomeric copies, or vaccines with CpG as an additional stimulator [139].

Another interesting, and much needed, area of research regards the development of vaccines against malaria, a significant neglected disease in many regions of the planet. This research area has also been using synthetic nanoparticles to build vaccines able to moderately protect against murine malaria in the blood [120]. Against malaria, adjuvants have to induce both antibodies and helper T cells; non-inflammatory polystyrene nanoparticles (PS NPs) as adjuvants and a protein conserved across several Plasmodium species named MSP4/5 as antigen, Th1 and Th2 immune responses was obtained; there was 50–80% protection against blood-stage malaria linked to interferon–gamma production [120]. Currently, no efficient vaccine against malaria in clinics is available; efforts to develop malaria vaccines have been reviewed [121,122,123]. Both discovery of better immunostimulatory formulations and adjuvants are needed. In addition, Toll-like receptors ligands that can increase immunity have also been considered important to be added as stimulators both in malaria [122,123] and cancer vaccines [140].

**Figure 9:**
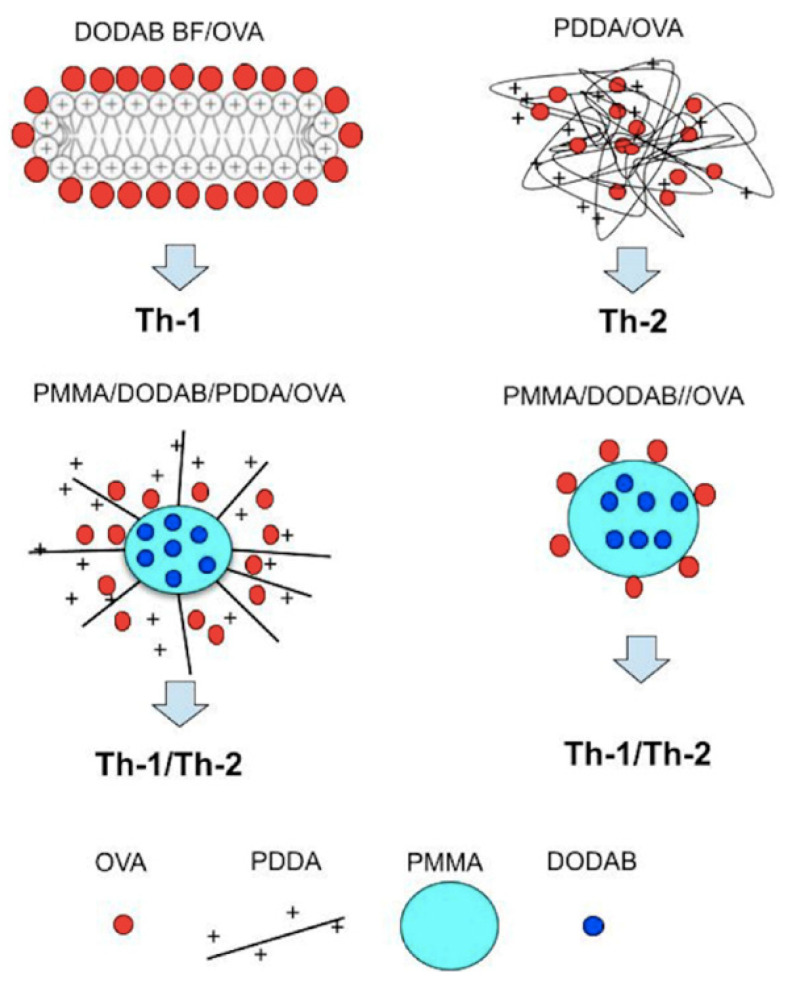
Nanoparticles (NPs) from cationic lipid and ovalbumin; cationic polymer and ovalbumin; biocompatible polymer, cationic lipid, cationic polymer, and ovalbumin; biocompatible polymer, cationic lipid, and ovalbumin. Reproduced from [46].

In our group we have been developing biomimetic nanoparticles for drug and vaccine delivery over the last 30 years [15,38,68,141,142,143,144,145]. Some review articles discussed the major role of novel cationic nanostructures based on polymers, lipids, and/or surfactants on efficient delivery of antigens to the immune system [3,4,12,69,146]. More recently, cationic nanoparticles based on biocompatible polymer PMMA [5,23,24,26,46,147] have also been developed besides the lipid nanodisks [16,17,18] and the supported bilayers [22,43,44]. In order to compare the immune responses elicited by the different cationic nanostructures first described in our laboratory while carrying ovalbumin (OVA), we recently made a scheme relating the nanostructure with the obtained immune response [46]. Figure 9 schematically shows a cross section of a DODAB bilayer fragment (DODAB BF) carrying OVA and inducing Th-1 response in mice [18], NPs of PDDA/OVA eliciting a Th-2 response (the hydrophilic PDDA cationic polymer combined with oppositely charged OVA yielding NPs) [92], NPs of PMMA/DODAB/PDDA/OVA eliciting a Th-1/Th-2 dual immune response [5], and NPs of PMMA/DODAB/OVA also yielding a dual Th-1/Th2 immune response [46]. The comparison between DODAB BF/OVA (Th-I inducer) and PDDA/OVA (Th-2 inducer) suggested that the interaction between DODAB BF and OVA inside the APC was weak and OVA readily desorbed from DODAB BF, reached the cytosol and was presented by MHC-I, whereas the interaction between PDDA and OVA in the APC was much stronger so that OVA could not so easily reach the APC cytosol remaining inside the endosome and being processed for presentation by MHC-II for enhancement of humoral immunity. OVA endosomal escape for Th-1 improved response was also obtained for two other cationic and biocompatible nanoparticles especially designed and synthesized from methylmethacrylate in the presence of DODAB or DODAB and PDDA to impart the cationic character to them; these NPs were mostly constituted of the biocompatible polymer PMMA and were named PMMA/DODAB/PDDA [5,147,148] and PMMA/DODAB NPs [23,26,46]. They could embed DODAB well mixed and distributed in the PMMA polymeric matrix [45] and displayed a core-shell structure when PDDA was also added to their synthesis: PMMA and DODAB in the core and PDDA as shell of PMMA/DODAB/PDDA NPs [5,24,147]. Importantly, macrophages and fibroblasts viability remained unaffected by DODAB and PDDA at the concentrations used in the NPs for testing their adjuvanticity in vivo [5,24,148]. Combined with OVA, NPs/OVA enhanced Th-1 and Th-2 responses [5,46]. Figure 9 displays some schematic cross-sections of four cationic adjuvants tested by our group along some decades; the type of response they induced is also quoted.

## 5. Hydrogels

Hydrogels made of synthetic and natural materials can also contain vaccines with the ability to modulate the immune response [149,150]. They impart a desirable sustained releasing property for the antigen/adjuvant able to enhance the humoral response [151].

**Figure 10:**
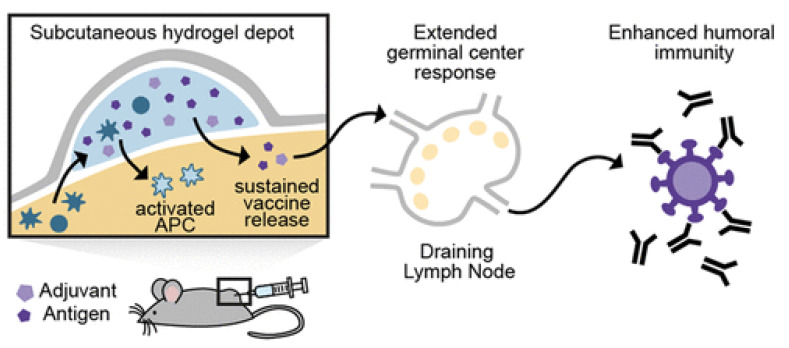
The concept of subcutaneous hydrogel depot for carrying vaccines. Adapted from [150].

Some interesting examples of hydrogels are those formed on the basis of the hydrophobic effect from polymerization of hydrophobic and hydrophilic monomers such as stearyl methacrylamide and acrylamide, respectively, in aqueous sodium dodecylsulphate dispersion; adding NaCl led to micelles growth; in another example, interactions that kept the hydrogel network were the hydrogen bridges between poly (vinyl alcohol) and the amino moiety of melamine; interestingly, in both hydrogels, deformations underwent self-healing, taking place by freezing and thawing the hydrogel under strain [151]. In a third example, a dodecyl-modified hydroxypropylmethylcellulose hydrogel interacted with poly (ethylene glycol)–b-poly(lactic acid) nanoparticles carrying OVA and poly(I:C) so that noncovalent interactions determined the cross-linking inside the polymeric hydrogel matrix; after injection of this hydrogel vaccine subcutaneously in mice, APC infiltrating the hydrogel became active and migrated to the LN; the result was sustained enhancement of OVA-specific humoral immune response; noncovalent interactions between the polymer and NPs were multiple physical cross-links within the hydrogel structure [150]. Figure 10 adapted from reference [150] illustrates the concept behind the sustained release behavior of vaccines formulated in hydrogels.

In an insightful work, considering that inflammation promotes cancer growth and suppresses immune responses against the cancer cells, peptides modified with anti-inflammatory moieties were used to obtain hydrogel carriers in the design of cancer vaccines; the incorporation of OVA in the hydrogel by vortexing hydrogel and OVA increased IgG and IgG2a production besides stimulating the secretion of IFN-γ and IL-6 cytokines in accordance with an enhanced dual immune responses of the humoral and cellular types [152]. In this respect, indomethacin is a nonsteroidal anti-inflammatory drug of hydrophobic nature, but bears a carboxylate in its chemical structure that could be well formulated with DODAB in aqueous dispersion to yield nanoparticles surrounded by an outermost layer of carboxymethyl cellulose, an hydrophilic biopolymer prone to be used in hydrogels [153].

Hydrogels can also respond to changes in temperature. Some of them are liquid at room temperature and change their state to gel at body temperature; they could be applied locally in tumors for cancer due to their sustained in situ delivery of therapeutic drugs, a very useful property for such thermo-responsive gels [154].

**Figure 11:**
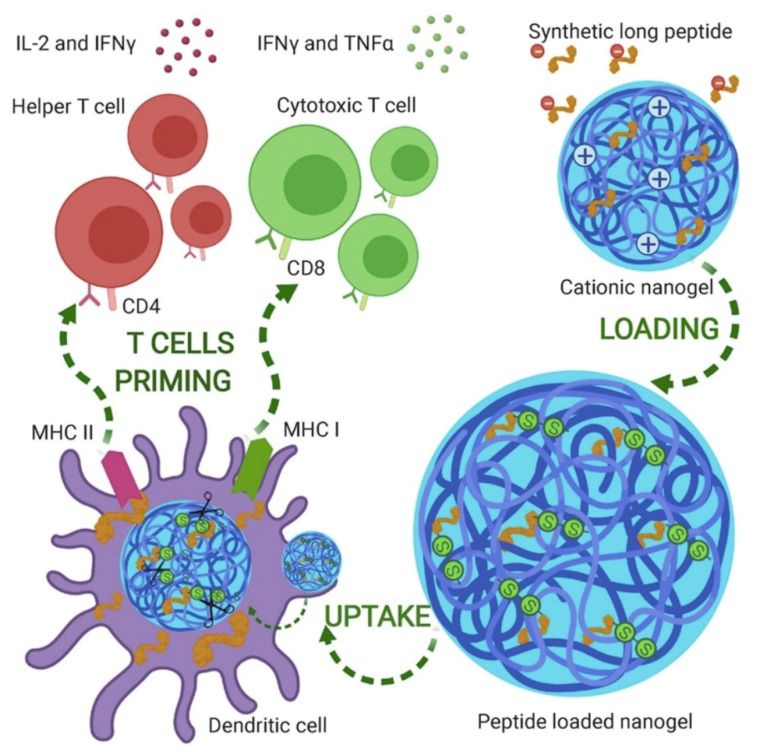
Positively charged gels developed as nanoparticles (sizes around 200 nm) used to carry peptides with epitopes for activating humoral and cellular immunity. Reproduced from [155].

Additionally applicable as therapy for cancer, some interesting nanogels shaped as nanoparticles were obtained from polymerization induced by ultra-violet light of [2-(methacryloyloxy)-ethyl] trimethylammonium chloride and dextran methacrylate in an oil-in-water emulsion; after loading these nanogels with appropriate peptides with or without cysteine moieties, there was an electrostatically driven adsorption or covalent binding of the peptides or cys-peptides to the nanogels, respectively; because the peptides had been synthesized with epitopes that induced maturation of dendritic cells, enhanced responses in terms of cellular immunity and cytokines secretion were also obtained; this was more so when the stimulator of Toll-like receptor poly(I:C) was also added to the formulation [155]. Figure 11 illustrates the cationic nanogels with applications for cancer nanovaccines in action [155].

## 6. Conclusions

This review is an overview on promising old and new biomimetic adjuvants for nanovaccines considering the requirements of nanometric size and biocompatibility. Nano systems such as bilayers fragments, disks or lipid nanoparticles, vesicles, liposomes, niosomes, nanoparticles, or hydrogels have been revealing their potential as adjuvants for decades, and indeed enhance immunity against important diseases affecting mankind.
